# Comparison between high‐resolution water‐perfused anorectal manometry and THD^®^ Anopress anal manometry: a prospective observational study

**DOI:** 10.1111/codi.14992

**Published:** 2020-02-21

**Authors:** C. A. Leo, E. Cavazzoni, M. M. N. Leeuwenburgh, G. P. Thomas, A. Dennis, P. Bassett, J. D. Hodgkinson, J. Warusavitarne, J. Murphy, C. J. Vaizey

**Affiliations:** ^1^ Sir Alan Park’s Physiology Unit St Mark’s Hospital Academic Institute Harrow UK; ^2^ Imperial College of London London UK; ^3^ The Royal London Hospital London UK; ^4^ Santa Maria della Misericordia Hospital Università degli Studi di Perugia Perugia Italy; ^5^ Haaglanden Medisch Centrum Den Haag The Netherlands; ^6^ Statsconsultancy Ltd Amersham UK

**Keywords:** Anopress, anorectal manometry, anorectal physiology tests, faecal incontinence, high‐resolution manometry, physiology, water‐perfused manometry

## Abstract

**Aim:**

Anorectal physiology tests provide a functional assessment of the anal canal. The aim of this study was to compare the results generated by standard high‐resolution water‐perfused manometry (WPM) with the newer THD^®^ Anopress manometry system.

**Method:**

This was a prospective observational study. Conventional manometry was carried out using a water‐perfused catheter with high‐resolution manometry and compared with the Anopress system with air‐filled catheters. All patients underwent the two procedures successively in a randomized order. Time to arrive at the resting pressure plateau, resting, squeeze, straining pressure and visual analogue scale (VAS) scores for pain were recorded. A qualitative analysis of the two devices was performed.

**Results:**

Between 2016 and 2017, 60 patients were recruited. The time from insertion of the catheter to arriving at the resting pressure plateau was significantly lower with the Anopress compared with WPM: 12 s [interquartile range (IQR) 10–17 s] versus 100 s (IQR 67–121 s) (*P* < 0.001). A strong correlation between the manometric values of WPM and the Anopress was observed. Both procedures were well tolerated, although the VAS score for insertion of the WPM catheter was significantly higher. The Anopress was easier to use and more time‐efficient than the WPM.

**Conclusion:**

The pressure values obtained with Anopress correlated well with those of conventional manometry. The Anopress has the advantage of being less time‐consuming, user‐friendly and better tolerated by patients.

**What does this paper add to the literature?**

This is a prospective observational study that shows the correlation between two manometric systems. To our knowledge this is the first paper to compare these two technologies. The Anopress seems to be easy to use and is also reliable and able to reproduce measurements of anal canal pressures, overcoming many of the limitations of water‐perfused manometry.

## Introduction

Functional disorders of the anus and rectum affect up to 20% of the population 1, 2, 3, 4. Anorectal physiology tests provide a functional assessment of the anal canal and are considered part of the standard of care for patients with pelvic floor disorders 4, 5, 6, 7. Currently, sphincteric assessment is performed by a combined approach of anatomical evaluation with endoanal ultrasound and functional assessment with anal manometry 5, 6, 7, 8, 9. One of the traditional methods for anal manometry involves multi‐channel water‐perfused catheters which take the average pressure at multiple intervals 10. Pressures are recorded after the catheter has been placed into the anorectum for a few minutes. Anal canal pressures are recorded while the patient is relaxed (resting pressures) and with the sphincter clenched (squeeze pressures) 11.

The THD^®^ Anopress (THD Worldwide, Correggio, Italy) is a new portable anal manometry device which uses air‐filled catheters to evaluate the sphincter pressures generated from the whole of the anal canal 12. The catheters have a pneumatic membrane and the machine has a topographical display to allow easier interpretation of data. It is promoted as a small, portable, wireless device that can perform rapid manometric assessment away from the anorectal physiology laboratory. This new technology provides manometric results from the whole anal canal at one time, giving a total passive and a total squeeze resistance 12, 13. Its normal values and ability to detect sphincter dysfunction have been demonstrated recently 14, 15.

If the Anopress proves to be as accurate as the current standard of care, it might be an attractive alternative. The aim of this study was to compare standard water‐perfused manometry (WPM) with the Anopress.

## Method

This prospective observational study to compare these two technologies received Health Research Authority approval by the London – Dulwich Research Ethics Committee prior to initiation (16/LO/1577). The study protocol was registered with the UK National Health Service National Health Research Authority under IRAS ID 207753 and the rationale was published 16.

### Patients

The study was performed in the Sir Alan Park’s Physiology Unit of St Mark’s Hospital, London, UK. Adult patients who were referred for anorectal physiology studies because of faecal incontinence in accordance with our national guidelines 17 were eligible for inclusion. Those referred for other reasons, such as anal pain, irritable bowel syndrome and inflammatory bowel diseases, were excluded from this study. Patients were approached and informed about the study in the anorectal physiology clinic by the doctor performing the tests. Full informed consent was gained prior to the tests. If patients declined or could not be included in this study they were offered anorectal physiology tests according to the current standard of care. We aimed to include 60 male and female patients in a 1:1 ratio for reasons of comparison between genders.

### Anorectal physiology studies

All tests were performed by two colorectal surgeons fully trained in both WPM and Anopress (CAL, JDH). Prior to the procedure, patients were instructed to defaecate if required and no bowel preparation was given. Investigators confirmed that all subjects understood the commands squeeze, cough and push prior to the procedures 18. All investigations were performed in the left lateral position in the presence of a chaperone. The manometric investigations were performed consecutively in the same clinic session. Patients were randomized with sealed envelopes to which technology was used first. All the standard manometry manoeuvres were performed according to the study protocols 16. After positioning of the catheter a period of 5 min was taken to allow for a resting pressure to be measured. Following this the maximum voluntary squeeze pressure, 5 s endurance, maximum involuntary squeeze and strain pressure were measured. Each manoeuvre was performed twice with a 30 s interval between each attempt.

### Water‐perfused anorectal manometry

**Figure 1:**
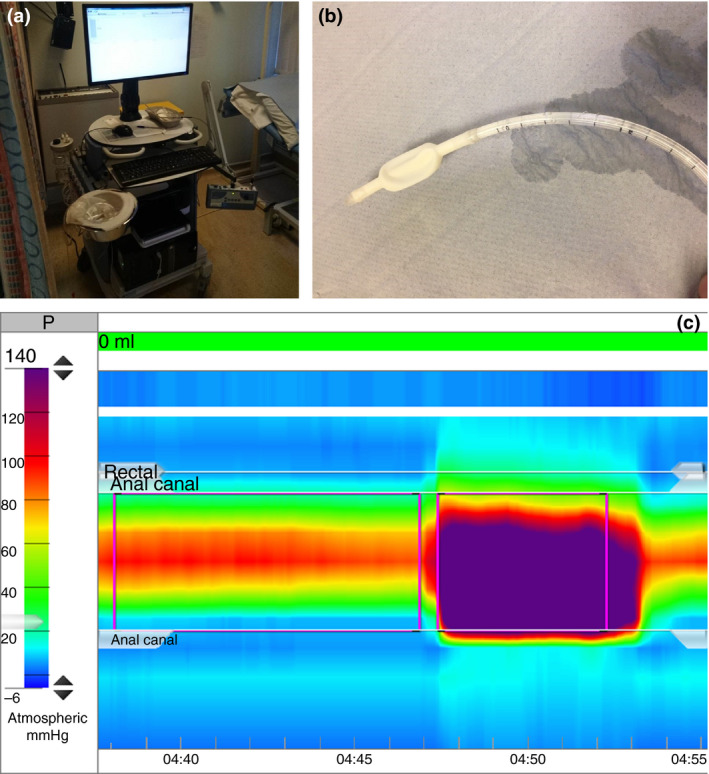
Water‐perfused high‐resolution manometry: (a) hardware device used in our centre for manometry and (b) water‐perfused catheter. (c) Pressures as they appear on the monitor for high‐resolution manometry.

Single‐use water‐perfused catheters were used; these had a diameter of 4.9 mm and 10 channels spaced at 8 mm intervals between 0 and 7.2 cm from the tip (Fig. 1a). The channels were connected to external pressure transducers which convert the received pressures to a high‐resolution colour plot displayed on the monitor of a solar MMS manometry system with High Resolution software, v.9.5 (Medical Measurements Systems, Enschede, The Netherlands) (Fig. 1b) 19. Prior to each test, catheters were infused with physiological solution and calibrated. The catheter was inserted into the anus to about 10 cm from the anal verge and advanced slowly until the balloon on the distal end of the catheter was in the rectum. This is represented on the display screen by a low‐pressure zone. A higher‐pressure zone representing the anal canal is in the centre of the graph and a further lower‐pressure zone representing the channels outside the body are seen on the monitor (Fig. 1c). Normal values used in clinic for this device are shown in Table 1.

**Table 1:** Normal values used in the Sir Alan Park’s Physiology Unit (St Mark’s Hospital) for water‐[perfused manometry (WPM) 11, 25 and normal values recently demonstrated for the THD^®^ Anopress 14.

	Female	Male
WPM (cmH_2_O)		
Maximum resting pressure	60–160	60–160
Maximum squeeze increment	50–180	60–220
Involuntary squeeze increment	50–100	50–100
Five seconds squeeze increment	40–160	40–200
THD^®^ Anopress (mmHg)		
Maximum resting pressure	40–103	38–100
Maximum squeeze increment	35–140	42–155
Involuntary squeeze increment	41–121	40–124
Ten seconds squeeze increment	44–98	43–103

### THD^®^ Anopress

**Figure 2:**
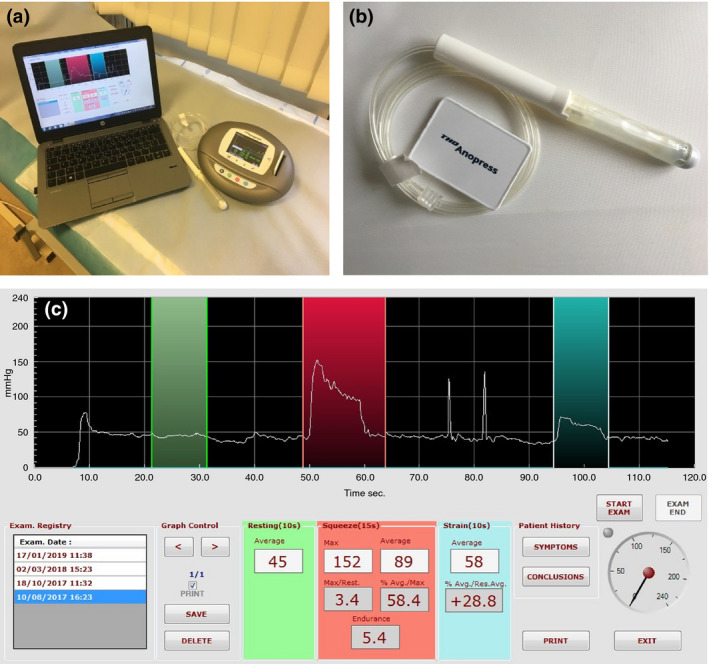
THD^®^ Anopress: (a) portable hardware device used for this study and (b) air‐filled catheters. (c) Pressure graphs as they appear on the Anopress screen.

Single‐use air‐filled catheters (THD PressProbe ENV, Fig. 2) measure the average pressure of the whole anal canal at once. This is achieved by a toroidal membrane in direct contact with the anal canal on one side and a pressure transducer on the other side 13. These allow pneumatic pressure measurements without the need for a sensor on the surface. These probes are 17 cm long, including 8 cm of pneumatic membrane, and have a maximum diameter of 15 mm. The catheter was calibrated prior to each test by allowing the air‐filled catheter to record the ambient atmospheric pressure. The catheter was then inserted into the anus for the entire length of the device. A minimum of 5 min was taken to stabilize the pressure. Then the same manoeuvres were repeated as with WPM above according to the study protocol 16. Normal values used in clinic for this device are shown in Table 1.

### Data collection

The investigating physicians prospectively recorded details of each patient’s medical history, physical examination and laboratory findings in a structured case record form as per standard care. St Mark’s faecal incontinence scores were obtained for all to allow objective measurement of symptom severity 20. A visual analogue scale (VAS) with 0 = ‘no pain at all’ and 10 = ‘my pain is as bad as it could possibly be’ was used at both insertion and during the procedures 21. The time taken for the resting pressure to stabilize, maximum anal resting pressure, main voluntary squeeze increment (difference between the maximum voluntary squeeze pressure and the maximum resting pressure), endurance in 5 s (increase of the average pressure within 5 s of maximum squeeze) and involuntary squeeze pressures for both tests were recorded.

### Statistical analysis

The resting and squeeze pressures were summarized by giving the mean and standard deviation if normally distributed, and the median with interquartile range (IQR) if not normally distributed. The aim of the analysis was to compare patient outcomes between the two test methods. For variables where the values were known to vary between methods, the analyses examined the strength of association between the patient outcomes of WPM and Anopress. Pearson correlation was used for normally distributed variables and Spearman’s rank correlation was used for variables not normally distributed. The correlation approach was felt to be the more appropriate methodology, because due to objective differences in the two devices we did not expect the same values from both. The results were displayed graphically in scatterplots. For other variables, the difference in values between methods was examined. Due to the repeated measurements per patient (one per method), the analyses were performed using multilevel regression methods. Multilevel linear regression was used for continuous outcomes and multilevel logistic regression was used for binary outcomes. The models included terms for the order in which the tests were performed, and any continuous outcomes found to exhibit a positively skewed distribution were given a log transformation before analysis. For the VAS score at insertion, a small constant of one was added before transformation as it was not possible to log transform zero values which were found for this variable. *P*‐values of < 0.05 were considered statistically significant. All analyses were performed using the software package Stata (v.15.1). Statistical methodology was undertaken by a professional medical statistician (PB).

### Qualitative analysis

The qualitative properties of both devices and catheters were detailed and compared in an objective way.

## Results

Between December 2016 and June 2017, 60 patients were recruited (30 men and 30 women) from the Sir Alan Park’s Physiology Unit at St Mark’s Hospital, UK. Their mean age was 58.6 years (SD ± 12.2 years). The mean St Marks faecal incontinence score was 14.6 (SD ± 5.9) out of 24. All patients received both tests. In 30 patients the WPM was performed first, and for the other 30 patients THD^®^ Anopress was performed first (Table 2). No complications or side effects were recorded during or after either test. The anorectal contractile reflex was observed in all patient. No difference was noted.

**Table 2:** Patient demographics and baseline characteristics. Summary statistics are: number (percentage) or mean ± standard deviation.

Variable	Category	Summary
Age (years)	–	58.6 ± 12.2
Gender	Male	30 (50%)
	Female	30 (50%)
Test order	WP first	30 (50%)
	THD first	30 (50%)
St Mark’s score	–	14.6 ± 5.9

### Manometric values

**Figure 3:**
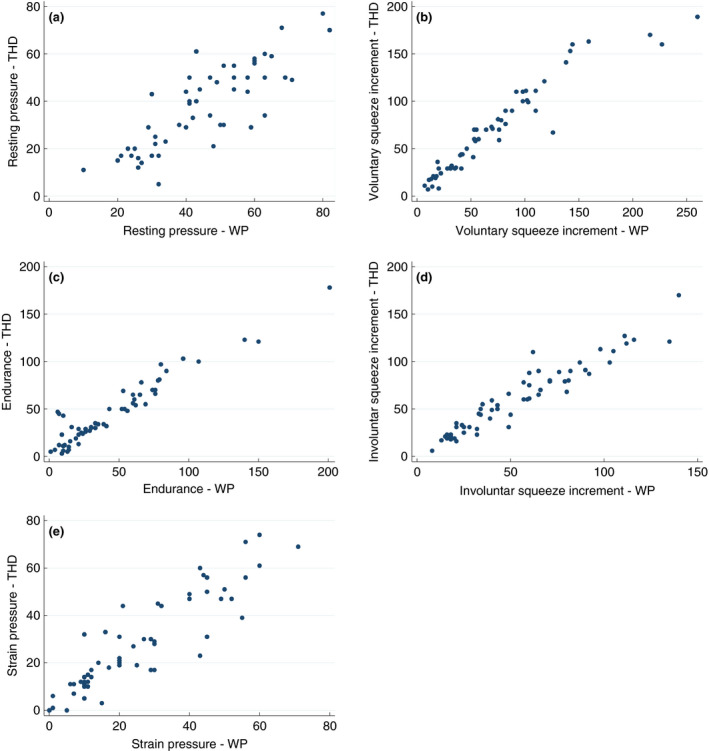
Graphical illustrations of the association between water‐perfused manometry (WPM) and Anopress are shown for (a) resting pressure measurements, (b) voluntary squeeze increment measurements, (c) endurance measurements, (d) involuntary squeeze increment measurements and (e) straining pressure.

The median time from insertion of the catheter to arrival at the resting pressure plateau was significantly lower in the Anopress compared with WPM; 12 s (IQR 10–17 s) and 100 s (IQR 67–121 s), respectively (*P* < 0.001). The WPM had a mean resting pressure of 44.3 cmH_2_O (SD ± 15.8 cmH_2_O), median voluntary squeeze increment of 57 cmH_2_O (IQR 31–102 cmH_2_O), mean squeeze endurance at 5 s of 43.7 cmH_2_O (SD ± 39.3 cmH_2_O), mean involuntary squeeze increment of 54.3 cmH_2_O (SD ± 33.1 cmH_2_O) and a straining pressure of 20 cmH_2_O (IQR 10–42 cmH_2_O). In comparison, the Anopress had a mean resting pressure of 37.5 mmHg (SD ± 17.0 mmHg), median voluntary squeeze increment of 64 mmHg (IQR 29–101 mmHg), mean squeeze endurance at 5 s of 44.7 mmHg (SD ± 35.1 mmHg), mean involuntary squeeze increment of 61.0 mmHg (SD ± 35.7 mmHg) and a straining pressure of 21 mmHg (IQR 11–45 mmHg). The correlation coefficients showed a strong positive association between the WPM and the THD^®^ Anopress measurements. These were 0.84 for resting pressure, 0.97 for voluntary squeeze increment, 0.90 for endurance of voluntary squeeze, 0.96 for involuntary squeeze increment and 0.91 for strain pressure (all with *P* < 0.001). All manometric values are listed in Table 3 and the associations between WPM and the Anopress are shown in Fig. 3.

**Table 3:** Comparison of outcomes between water‐perfused manometry (WPM) and THD Anopress, giving the median (interquartile range, IQR) or mean ± SD and the correlation between measurements and *P*‐values indicating the significance of the association.

Measurement	THD^®^ Anopress	WPM	Correlation coefficient	*P*‐value
Time to resting pressure	12 s (IQR 10–17 s)	100 s (IQR 67–121 s)	NA	<0.001
Resting pressure	37.5 ± 17.0 mmHg	44.3 ± 15.8 cmH_2_O	0.84*	<0.001
Voluntary squeeze increment	64 mmHg (IQR 29–101 mmHg)	57 cmH_2_O (IQR 31–102 cmH_2_O)	0.97†	<0.001
Endurance at 5 s	44.7 ±35.1 mmHg	43.7 ±39.3 cmH_2_O	0.90†	<0.001
Involuntary squeeze increment	61.0 ±35.7 mmHg	54.3 ±33.1 cmH_2_O	0.96†	<0.001
Straining pressure	21 mmHg (IQR 11–45 mmHg)	20 mmHg (IQR 10–42 mmHg)	0.91†	<0.001

* Analysis using Pearson correlation (normally distributed).

^†^ Analysis using Spearman’s rank correlation (not normally distributed).

Additionally, as part of the same analysis the differences in outcome were assessed between the two periods (first test versus second test). The results suggested that none of the outcomes varied significantly between the two study periods. There were also no statistically significant differences in outcome when comparing the results between the two genders.

### Patient tolerance of the devices

The median VAS score at introduction of the catheter for the WPM test was significantly higher (VAS of 2, IQR 1–5) than for the Anopress, which had a median VAS score of 0 (IQR 0–1; *P* < 0.001). The procedure was tolerated well for both groups. The VAS score was mostly zero for both techniques. Eleven out of 60 (18%) patients reported discomfort during the WPM test with a VAS score higher than 1. Seven out of 60 (11%) reported discomfort during the THD^®^ Anopress test (*P* = 0.31).

### Qualitative analysis

Details of the qualitative analysis are listed in Table 4. The WPM is heavy and bulky compared with the Anopress. The latter is portable, so was felt to be easier to use than WPM when there was no assistant to help. The Anopress catheter requires a maximum of 10 s to calibrate 14. This is an advantage when compared with the greater length of time needed to calibrate the catheter of the WPM 10. The WPM catheters used are thinner (4.9 mm), but long and floppy. They require the catheter to be passed into the rectum to allow the channels to gain position in the anal canal. The Anopress probes are slightly thicker (15 mm) but are ergonomic and have a smoother surface. They simply require passage into the anal canal to achieve a correct position. Conversely, the WPM allows measurement of the length of the anal canal and has an integrated rectal balloon test, neither of which are possible with the Anopress probes used for this study.

**Table 4:** A qualitative analysis of the two devices: THD^®^ Anopress and water‐perfused manometry (WPM). The top rows describe the two main devices and software. The bottom rows describe the two catheters used for this study.

	WPM	THD® Anopress
Body		
Weight and size	Heavy and bulky	Light
Connection	Wired	Wireless
Motility	Stationary	Portable
Software	High resolution	Coloured single trace
Reading	Coloured graph	Linear plot
Cost*	High	Low
Catheters		
Technology	Water perfused	Air filled
Preparation	Time‐consuming	Easy and quick
Calibration	3–5 min	5–10 s
Sensors	Ten spaced sensors	Pneumatic membrane
Technique	Stationary	Pull‐through examination
Position	Anorectum	Anal canal
Length of the anus	Accurate	NA
Balloon tests	Possible	NA
Test lifespan	Long	Short
Cost*	High	Cheap

* Note: cost comparison is based on the UK market. This may vary from country to country and by order volume. The price indicated by the two companies is £4950 (Anopress device) *vs* £18 000 (WPM device). An Anopress catheters costs £22–40 *vs* £50–60 for WPM.

## Discussion and conclusions

This prospective observational study showed a strong correlation between the manometric measurements of the current standard of care, high‐resolution WPM, and the newer Anopress. The Anopress arrived at the resting pressure plateau significantly faster than the water‐perfused catheter and the qualitative analysis shows that the Anopress may also have overcome many of the disadvantages of WPM.

Firstly, the Anopress is smaller, lighter, portable and wireless, and was deemed to be more user‐friendly than WPM. Secondly, the preparation and calibration of the catheter is easier and faster. Furthermore, the Anopress device is much cheaper than the WPM (£4950 *vs* £20 000) and the catheters are cheaper on average (£22 *vs* £40). The Anopress single‐line plot manometry may be easier to read, which may make it simpler to train staff to perform these investigations. The Anopress measures the anal canal as a whole, and this may mean less inter‐user variability compared with WPM, the values for which may vary significantly between users; therefore the Anopress may offer a better correlation with the symptomatology of patients compared than WPM because it evaluates the sphincter as a whole. In comparison, WPM provides only maximal resting and squeeze pressures generated by an isolated area of a few millimetres in the anal canal 22. This may disregard the contribution of the rest of the muscle complex 23 and it could explain why there has been a poor correlation between manometry and the symptomatology of individual patients with faecal incontinence. Patients can have a structurally intact sphincter with normal manometry values and be symptomatic. Others may have sphincter defects and attenuated manometry and be symptom‐free. Further studies are needed to investigate this observation. Lastly, the Anopress was better tolerated by patients, who reported lower scores for pain and discomfort: this perhaps arises from the design of the two respective catheters and from the different position of the catheters during the test: WPM have catheters in the anorectum, Anopress are positioned in the anal canal only.

The THD^®^ Anopress catheters were found to have a few limitations. These included the inability to measure the length of the anal canal, the rectoanal inhibitory reflex and rectal capacity. However, it is recognized that the length of the anal canal may not be clinically relevant 24 and its measurement is unnecessary, especially if endoanal ultrasonography is used as a correlated test 17. Balloon tests were not possible using the Anopress catheters. However, whilst assessment of rectal capacity and the balloon expulsion test are essential parts of anorectal physiology tests they are not considered manometric tests. Therefore, the comparison was not necessary and not required. This limitation has been addressed by the currently available catheters that have a balloon at the tip, but these were not available at the time of this study.

This study was limited to only two specific products, whereas numerous catheters, technologies and software are available commercially. However, many of the advantages and disadvantages of both technologies are known to be relatively common between the current technologies. Another limitation of this study was lack of a sample size calculation; this was not achievable because the published data on Anopress are scarce and did not provide sufficient information to perform this calculation. Furthermore, the sample size was limited to 60 patients by the medical ethical committee before this study started. We did not observe differences when comparing results between the two genders. The specified sample of 30 men and 30 women could introduce an obvious selection bias which could be discarded by the fact that all patients underwent both investigations in a randomized order.

In conclusion, the Anopress measurements correlate strongly with WPM. It is also a user‐friendly, time‐efficient device and it appears to be better tolerated than standard WPM.

We do not think the Anopress should entirely replace the WPM; however, we feel that the Anopress should be accessible to all units with an interest in pelvic floor surgery. There may be associated cost savings when compared with current practice.

## Financial support

None.

## Conflicts of interest

None.

## Author contributions

CAL and EC contributed to design of the study, acquisition of data, analysis and interpretation of data and drafting/revising the manuscript critically for important intellectual content; MMNL, GPT, JH, AD, JW and PB have contributed to the design and interpretation of data and revising the manuscript; JM and CJV have contributed to the conception and design, acquisition of data, analysis and interpretation of data, drafting/revising the manuscript critically for important intellectual content; all authors provided a final approval of the version to be published.
